# The King of Sweden and M. Gama

**Published:** 1836-01

**Authors:** 


### THE KING OF SWEDEN AND M. GAMA.

The King of Sweden, through his ambassador, has just presented to M. Gama, (chief surgeon of the Val de Grace,) the title of Chevalier of the order of Vasa, together with a decoration enriched with diamonds. A short time before his departure for Sweden, the king, then prince of Ponte-Corvo, was making a reconnaissance of the enemy's positions, when he was struck by a ball behind and above the mastoid process. M. Gama, surgeon-major of the Avant-garde, was immediately called, and joined the wounded prince in a farm-house, who was giving the necessary orders for the care of his troops, despatching the officers in all directions, and dictating a letter to the emperor. When this was concluded, the prince addressed himself to M. Gama, who dressed the wound after making a necessary incision, and offered him the attendance of one of his surgeons: the prince, however, opposed this, not wishing to take any one from his post, and recommended M. Gama "to go, and give the others their turn." This circumstance, which M. Gama has recently mentioned in his treatise on Wounds of the Head, has refreshed the recollection of the king of Sweden, who immediately sent to the author this honourable proof of his remembrance of his services.

*Gazette Médicale de Paris*, No. 43, Octobre 24, 1835.

